# Ohio State Dental Society

**Published:** 1886-11

**Authors:** 


### EDITORIAL.

### Ohio State Dental Society.

The annual meeting of this body has just been held, and was successful beyond the highest expectation of its most ardent friends. About thirty new members were elected. There were about one hundred persons present. There were present quite a goodly number from all the adjacent States.

The organization and its working is most perfect, but little time being devoted to routine work in the general meeting.

The meeting was in every respect satisfactory. The arrangements provided by the Executive Committee were the best the Society has ever had, and if such an Executive Committee could always be had, there would be no fear about good meetings. We will anticipate just as good a meeting next year, for the place selected is central—Springfield; and Dr. Runyan is at the head of the committee, and everybody should attend and see how things are done.

Five candidates were before the State Board of Examiners, four of whom received the approval of the Board.

A full report of the proceedings and discussions, and some of the papers at least, will appear in the December REGISTER; these, we think, will be found worthy of careful reading.

